# Trace element contents in toenails are related to regular physical activity in older adults

**DOI:** 10.1371/journal.pone.0185318

**Published:** 2017-10-06

**Authors:** Antoni Sureda, Maria del Mar Bibiloni, Alicia Julibert, Raquel Aparicio-Ugarriza, Gonzalo Palacios-Le Blé, Antoni Pons, Marcela Gonzalez-Gross, Josep A. Tur

**Affiliations:** 1 Research Group on Community Nutrition and Oxidative Stress, University of the Balearic Islands, Palma de Mallorca, Spain; 2 CIBEROBN (Physiopathology of Obesity and Nutrition CB12/03/30038), Instituto de Salud Carlos III, Madrid, Spain; 3 ImFINE Research Group, Department of Health and Human Performance, Faculty of Physical Activity and Sport Sciences (INEF), Technical University of Madrid, Madrid, Spain; Chinese Academy of Sciences, CHINA

## Abstract

The aim was to assess the trace element contents in toenails of older adults and its association with regular physical activity. Cross-sectional multicentre study in Spain, collecting data from a random sample of 380 participants (54% female) aged 55–80 years (men) and 60–80 years (women) with no previously documented cardiovascular disease. Physical activity performed was measured using the Minnesota Leisure-time Physical Activity Questionnaire. The 25 most inactive and 25 most active individuals for each sex were selected for this study (final sample *n* = 100). Anthropometric measurements were performed and toenail samples collected for calcium (Ca), chromium (Cr), iron (Fe), cobalt (Co), nickel (Ni), zinc (Zn), selenium (Se) and mercury (Hg) analysis. Significant differences between sexes were reported in Ca concentrations, women having lower concentrations than men. No differences were reported in trace element contents between active and inactive men. Active women showed higher Ca, Cr, Fe, Co, and Zn and lower Hg contents than their inactive peers (all p<0.05). Inactive women showed lower Ca and Co levels (735.0 mg/kg and 4.5 μg/kg, respectively) than inactive men (1170.0 mg/kg and 7.9 μg/kg, respectively). Active women had lower Ca and higher levels of Cr (936.0 mg/kg and 1230.0 μg/kg, respectively) than active men (1070.0 mg/kg and 522.0 μg/kg, respectively). The present data added new information on the element contents in toenails of healthy Spanish older adults. The concentration of trace elements was similar in both sexes except for Ca which were lower in women. The trace element contents in women’s toenails, but not in men, were markedly influenced by physical activity, with higher levels of Ca and Fe and lower Hg among active females.

## Introduction

The ageing process is characterized by a progressive accumulation of molecular and cellular damage that can be modulated by environmental exposure, stress, lifestyle and nutrition [1]. Sarcopenia and osteoporosis are key factors implicated the progression of disability with ageing. The progression of these signs of fragility can be counteracted by the combination of adequate nutritional intake and exercise training [2].

Regular physical activity prescription for healthy ageing is a key point for chronic disease management and prevention [3]. Old adults performing high physical activity have greater conservation of lean tissue with reduced levels of sarcopenia than individuals who are more sedentary [4]. Moreover, regular exercise could protect bone and cartilage against osteoporosis by regulation of body trace elements involved in the biosynthesis of bone matrix structures and inhibition of bone resorption process [5]. However, mineral intake can be reduced in the elderly as a consequence of malnutrition, reduced capability of digestion and absorption or interaction with medication [6].

Trace elements are necessary for normal functioning of a variety of biological processes and any deficit could alter metabolic pathways including inflammatory and oxidative processes [7]. Monitoring the trace element concentrations in the human body is of critical importance for health and longevity. In fact, trace element concentration profiles in the body have been reported to be correlated with longevity in healthy people over 80 years when comparing longevous region vs. non-longevous regions [8].

Heavy metals such as mercury are highly toxic elements known to cause DNA damage, lipid peroxidation and protein oxidation and deactivation and are also associated with cardiovascular diseases [9–11]. Exposure to cadmium has been associated with decreased bone density [12]. The trace elements concentration in metabolically inactive fingernails has been used to monitoring the exposure to toxic metals [13–15] or to assess associations between excess or deficiency of trace elements and risk of cognitive decline in ageing [16], chronic kidney diseases [17], metabolic syndrome [18], inflammation [19], amyotrophic lateral sclerosis [20], chronic depression [21], cancer risk [22–24], or cardiovascular diseases [25].

The mineral components of nails mainly derive from the blood circulating pool and may reflect the long-term patterns of mineral metabolism reflecting a long-term period of 12–18 months [26,27]. The nail’s mineral content of individuals varies between populations, and not all minerals are deposited on the nails at the same speed [28]. The different elements present in nails have different metabolic roles in the body, and consequently, they could be used as indicators of their nutritional status. Moreover, the trace metal content in nails following physiological processes is practically unknown as well as their role in the mineral metabolism. Evaluating trace element concentrations in nails could be a good and non-invasive method to study the nutritional status individuals. Also, toenail are useful because they are environmentally sheltered in populations that wear shoes; they are less prone to superficial contamination than hair; and they are easily collected, transported, stored and cleaned [29]. Then, the aim of the present study is to assess the trace element contents in the toenails of older adults and its association with regular physical activity.

## Materials and methods

### Study population, design and ethics

The present study was a cross-sectional multicentre study (Balearic Islands and Madrid, Spain, 2013–2014), collecting data from a random sample of 380 participants (54% female) aged 55–80 years (men) and 60–80 years (women) with no previously documented cardiovascular disease. The exclusion criteria were to be institutionalized, suffering from a physical or mental illness that would have limited their ability to respond to the questionnaires, or drug intake for clinical research. The study was conducted according to the guidelines laid down in the Declaration of Helsinki, and all procedures were approved by the Ethics Committee of the Technical University of Madrid. Written informed consent was obtained from all participants.

The physical activity performed by the participants was measured using the Minnesota Leisure-time Physical Activity Questionnaire previously validated for the Spanish old adult population [30,31]. This questionnaire included a list of physical activities and the participants were asked about what type of leisure-time physical activities (LTPA) they had performed during the last year. Then, the participants estimated the duration of the activities performed in min/week, and then the participants were classified by the number of METmin/week. In order to assess the association between the trace element contents and regular physical activity, the 25 most inactive and 25 most active individuals for each sex were selected (final sample *n* = 100). According to the zinc concentration (mean: 107.5 mg/kg; IC95%: 104.5–110.7) in toenails reported by Martin-Moreno et al. 2003 [27], in 724 men (53.2 ± 9.3 years) from eight European countries and Israel without a history of myocardial infarction, the margin of error of the present sample size (n = 100), with a 95% confidence interval, was 7.3 mg/kg.

### Body composition

Anthropometric measurements were performed by well-trained observers in order to minimise the inter-observer coefficients of variation. Height was determined using a mobile anthropometer (Seca 213, SECA Deutchland, Hamburg, Germany) to the nearest millimetre, with the subject’s head in the Frankfurt plane. Body weight, body fat and muscle mass were determined using a Segmental Body Composition Analyzer (Tanita BC-418, Tanita, Tokyo, Japan). The participants were weighed in bare feet and light clothes, and subtracting 0.6 g for their clothes. Waist circumference (WC) was measured as the smallest horizontal girth between the costal margins and the iliac crests at minimal respiration using a flexible, non-extensible plastic tape with 0.1 cm precision (Kawe 43972, Kirchner & Wilhelm GmbH + Co. KG, Asperg, Germany). Weight and height measures were used to calculate body mass index (BMI, kg/m^2^). WC and height measures were used to calculate waist-to-height ratio (WHtR).

### Trace element analyses

Participants were instructed to collect at least 25 mg of toenails in a specific plastic bag. Women had to put off the nail polish before cutting the nails. The toenail samples were carefully cleaned in acid-washed beakers where they were cleaned sequentially with acetone, distilled deionized water (3 washes), and acetone again in a sonic bath. The samples were then placed in a drying oven for 2 hours at 50°C, after ensuring a constant weight. The dried samples were then places into polyethylene capsules ready for analysis. Toenail samples were collected for the analysis of calcium (Ca), chromium (Cr), iron (Fe), cobalt (Co), nickel (Ni), zinc (Zn), selenium (Se) and mercury (Hg). The concentration of trace elements in the fingernails was assessed using Instrumental Neutron Activation Analysis (INAA) at the Interfaculty Reactor Institute at Delft University of Technology in Delft (The Netherlands), following analytic methods and validation of these methods described by Blaauw [32]. The laboratory has an implanted quality control system for quality assurance and management, which complies with the requirements of the International Standard ISO/IEC 17025:1999 and has been accredited by the Dutch Council for Accreditation since 1993. Quality control of the data was performed using standard reference material NBS1577b from bovine liver (National Institute of Standards and Technology, Gaithersburg, MD, USA) in addition to procedural blanks. In each series of samples, reference materials were incorporated, and a blank capsule was analysed along with the rest to safeguard against trace element contamination in the analysis process. The results obtained from blanks and reference materials are compared to the previous results according to the Dutch guideline NPR 6603. The coefficient of variation (CV), as an expression of inter-assay variation for the tested metals was <5% for reference material. The intra-assay CV of Ca, Cr, Fe, Zn, Se and Hg concentration in toenails was <10%, and the CV of Co and Ni concentration was 10–15%.

### Statistical analysis

Analyses were performed with the SPSS statistical software package version 24.0 (SPSS Inc., Chicago, IL, USA). Since physical activity is usually different between men and women, all analyses were stratified by sex. Normality of data was assessed using Shapiro-Wilk test. Difference in means between two comparison groups were tested by unpaired Students’ *t*-test when variables were normally distributed, and difference in medians between two comparison groups were tested by the Mann-Whitney U test when variables were not normally distributed. An aligned rank test for a nonparametric analysis of the two way interaction was used [33–35]. A p value ≤0.05 was considered statistically significant.

## Results

Characteristics of study participants are shown in Table 1. In women, no significant differences were reported between active and inactive groups except for the degree of physical activity. Active men had lower weight, BMI, body fat and WHtR, and higher METmin/week than inactive peers.

**Table 1:** Characteristics of the participants.

	Men (*n* = 50)		Women (*n* = 50)	
	Inactive (*n* = 25)	Active (*n* = 25)	Inactive (*n* = 25)	Active (*n* = 25)
Age (years)	64.8 ± 1.2	65.0 ± 1.2	68.2 ± 1.0	66.8 ± 0.9
Weight (kg)	85.1 ± 2.5	73.5 ± 2.0*	66.5 ± 1.8	65.5 ± 2.0
Height (cm)	170.9 ± 1.3	168.1 ± 1.1	156.3 ± 1.0	155.7 ± 0.9
BMI (kg/m^2^)	29.0 ± 0.6	26.0 ± 0.7*	27.2 ± 0.7	27.0 ± 0.8
Body fat (%)	28.4 ± 0.8	23.2 ± 1.1*	38.2 ± 0.9	36.2 ± 1.2
WC (cm)	101.5 ± 1.8	89.6 ± 2.2*	86.3 ± 1.4	83.7 ± 1.8
WHtR	0.59 ± 0.01	0.53 ± 0.01*	0.55 ± 0.01	0.54 ± 0.01
Physical activity (METmin/week)	1561 ± 116	9764 ± 287*	1667 ± 133	8143 ± 334*

Abbreviations: BMI, body mass index; WC, waist circumference; WHtR, waist-to-height ratio.

Values are presented as mean ± SEM. The significance of the differences in these factors between inactive and active participants was tested by using unpaired t-test (* indicates p<0.05)

Trace element contents in toenails of men and women are presented in Table 2. Women showed lower concentrations of Ca respect men. No other differences were observed between sexes.

**Table 2:** Comparison of trace element contents in toenails of males and females.

	Men			Women			*P*
	*n*	Median	IQR	*n*	Median	IQR	
Ca (mg/kg)	50	1090.0	763.0–1412.5	50	832.5	580.8–1120.0	0.013
Cr (μg/kg)	50	554.0	333.8–1247.5	50	793.0	356.0–1630.0	0.238
Fe (mg/kg)	50	14.0	9.2–25.7	50	11.8	7.8–20.4	0.133
Co (μg/kg)	50	10.6	4.5–22.2	50	6.6	4.1–15.3	0.183
Ni (μg/kg)	46	989.0	567.8–2032.5	43	887.0	360.0–1660.0	0.352
Zn (mg/kg)	50	107.7	96.6–115.0	50	105.0	95.9–120.0	0.858
Se (μg/kg)	49	571.0	521.5–625.0	50	582.5	543.8–670.3	0.361
Hg (μg/kg)	50	549.0	292.5–942.5	50	610.5	462.3–888.0	0.260

Abbreviations: calcium (Ca); chromium (Cr); iron (Fe); cobalt (Co); nickel (Ni); zinc (Zn); selenium (Se); mercury (Hg); IQR, interquartile range. The significance of the differences between sexes was performed by Mann-Whitney U test.

Trace element contents in active/inactive men and women are showed in Table 3. No differences were reported in trace element contents between active and inactive men. However, active women showed significant higher Ca, Cr, Fe, Co, and Zn and lower Hg contents than inactive peers (all p<0.05). When analysing differences between sexes, inactive women reported significant lower Ca and Co levels than inactive men. Active women had lower Ca and higher levels of Cr than active men. Finally, the interaction between sex and physical activity (active vs. inactive) on each of trace mineral levels shown statistical significant differences in Cr (F = 12.650, p = 0.001), Fe (F = 4.436, p = 0.038), Co (F = 51.267, p<0.001) and Zn (F = 5.139, p = 0.026); whereas statistical significant differences were not found in Ca (F = 0.001, p = 0.978), Ni (F = 0.957, p = 0.957), Se (F = 0.140, p = 0.709) and Hg (F = 0.217, p = 0.642).

**Table 3:** Trace element contents in toenails of inactive and active men and women.

	Inactive			Active			
	*n*	Median	IQR	*n*	Median	IQR	P*
Men							
Ca (mg/kg)	25	1170.0	714.5–1365.0	25	1070.0	766.5–1425.0	0.938
Cr (μg/kg)	25	578.0	411.0–143.0	25	522.0	267.0–1125.0	0.277
Fe (mg/kg)	25	13.1	7.5–22.3	25	15.8	9.5–34.3	0.174
Co (μg/kg)	25	7.9	3.9–14.0	25	14.1	5.9–30.1	0.061
Ni (μg/kg)	23	1070.0	692.0–2250.0	23	769.0	464.0–2075.0	0.590
Zn (mg/kg)	25	106.0	90.8–114.0	25	108.0	100.0–115.5	0.351
Se (μg/kg)	25	595.0	526.0–677.5	24	563.5	521.3–596.3	0.136
Hg (μg/kg)	25	604.0	322.0–962.5	25	514.0	266.5–965.0	0.607
Women							
Ca (mg/kg)	25	735.0†	509.5–885.0	25	936.0	722.5–1310.0	0.050
Cr (μg/kg)	25	482.0	229.0–1365.0	25	1230.0†	535.5–3090.0	0.015
Fe (mg/kg)	25	8.8	6.5–13.5	25	14.1	8.9–27.8	0.010
Co (μg/kg)	25	4.5†	3.0–6.1	25	14.7	9.8–33.2	<0.001
Ni (μg/kg)	22	885.0	534.8–1425.0	21	887.0	297.0–1860.0	0.902
Zn (mg/kg)	25	97.0	90.1–111.0	25	110.0	103.0–123.5	0.012
Se (μg/kg)	25	577.0	534.5–680.0	25	588.0	549.5–387.0	0.861
Hg (μg/kg)	25	735.0	522.5–1065.0	25	555.0	387.0–695.0	0.010

Abbreviations: calcium (Ca); chromium (Cr); iron (Fe); cobalt (Co); nickel (Ni); zinc (Zn); selenium (Se); mercury (Hg); IQR, interquartile range.

*The significance of the differences between inactive and active participants within each sex was performed by Mann-Whitney U test.

† The significance of the differences between inactive and active participants between each sex was also performed by Mann-Whitney U test.

## Discussion

The present data attempts to establish the baseline range of trace elements in the toenails of older adults. Trace elements exert many functions in the body to ensure the normal metabolism and homeostasis. Deficiencies and excesses in any of these elements may contribute to the appearance and/or progression of several diseases [36–38]. For example, elevated or reduced levels of some metals such as Fe, Cu, Zn or Se have been reported to act as indicators of depression in young people [39]. Accordingly, analysing and monitoring trace elements in body tissues could be useful biomarkers in the diagnostic or in the follow-up of these diseases. However, the contents of the chemical elements vary in a broad range [40]. The wide concentration range of often several orders of magnitude may be explained by individual particularities of a person, but also to geographical differences, nutritional status and environmental conditions [28]. In addition, the associations between the blood concentrations of elements and its tissue concentrations are still unclear, and even more the relation with the concentrations found in the nails.

It has been evidenced that four characteristic elements including Cr, Fe, Mn, and Co are closely related to health in old people; their concentrations significantly increased in 80 years healthy old people living in longevous regions [8]. Nevertheless, a previous study performed with centenarians reported lower concentrations of trace elements than the results reported by other authors analysing younger populations [38]. These authors suggested that, in general, the concentrations of trace elements in nails decreases with ageing, although metals associated with heavy metal pollution depends more on the exposure. Progress in cognitive impairment among elderly population is associated with increased levels of Cu and Mn in fingernails [16]. The contents of these elements in nails of elderly people are related to those in drinking water or dietary intake [7].

In previous studies analysing nail trace elements in patients with amyotrophic lateral sclerosis, melanoma and controls in the Mediterranean region of Emilia-Romagna (northern Italy), the concentrations from controls were in the range to the measured in the present study [20,41]. In another study performed with Spanish healthy young adults, the nail concentrations of Zn and Se were also similar to our results [19]. In a European multi-centre case-control study on antioxidants, which found no association of toenail Zn with the risk of a first myocardial infarction, the Zn values reported [27] were closely similar as our results. Finally, non-exposed population from Panasqueira mine area (Portugal) reported values on Cr and Ni in the range of the present data [42]. The results from this and other studies performed in Mediterranean populations [43] showed similar trace element concentrations suggesting that people from these areas are under similar geographical and environmental conditions and also share similar nutritional habits.

The only differences evidenced between sexes were in Ca concentrations, higher in men than in women, which is in agreement with previous findings [28]. Ca is the most important mineral in bone metabolism, and a low Ca intake is associated with a reduced bone mass and osteoporosis [44]. In fact, positive correlations exist between Ca concentration in women’s toenails and fingernails and lumbar bone mineral density [45]. Toenail and fingernail Ca concentrations decrease significantly with age in men and women from 20 to 80 years old [46], and a progressive decrease in plasma Ca concentration associated with age, especially in women, has also previously been reported [44]. This decrease probably reflects the gradual decrease in Ca absorption with age, especially in post-menopausal women as a consequence of a decreased estrogen production [46,47]. This trend could be reflected in the incorporation Ca in toenails, as we evidenced with lower values in aged women respect to men of the same age.

Regular exercise may increase micronutrient requirements, either by increasing degradation rates or by increasing losses from the body. In this sense, elements such as Se, Zn, Fe, and Ca can potentiate the immune function and Ca and Fe contribute to muscle and heart contraction and relaxation which are central points for athletes or active people [48]. An adequate balance in trace elements is essential for health since and trace elements can be potential risk factors for many diseases. In a previous study, exercise and an adequate balance in serum Zn and Cu levels, have been reported to exert beneficial effects in improving depressive status in children and adolescents [49]. However, trace element deficiencies, in practice, are generally uncommon with the possible exceptions of Fe and Ca [50]. The present results reported no association between physical activity and any element content in men; on the contrary, active women showed higher concentrations of Ca, Cr, Fe, Co, Zn and Hg. Physical activity level has been associated with food choice, with higher appearance of cereals, fruits and vegetables in the diet of active participants [51]. It was previously reported that sedentary and low-active participants showed the lowest adherence to the Mediterranean dietary pattern [52]. In addition, a higher Ca intake was found in post-menopausal former athletes compared to non-athletes [53]. The higher levels of these elements in active women could be related with a healthy diet.

Se [53] and Zn [27] values in this study are similar to those reported in Europe, but Zn values were higher than other findings in toenails of Europeans [54]. Se and Zn have an antioxidant function by forming part of the glutathione peroxidase and superoxide dismutase enzymes, respectively [55]. Toenail Zn levels (reflecting long-term dietary intake) were not significantly associated with acute myocardial infarction risk [55]. Although, no significant differences were reported in Se and in Zn in men, a trend to increase was evidenced suggesting a better antioxidant profile in people practicing more physical activity. Accordingly, it has been reported that participants with a lower serum Se concentration may be exposed to a greater chronic oxidative stress due to neutrophil reactive oxygen species production [56].

The concentration of Hg measured in the toenails of our population is within the range than the values obtained in other studies performed with European participants [42,43,57], but higher than other findings in Ireland [54]. Hg is a toxic metal and its exposure can show a significant threat to human health with potential deleterious effects on the nervous and renal systems. The main form of Hg exposure in a Mediterranean population is from seafood due to the great bioaccumulation of metals through the food chain [58]. Interestingly, in the present study, more active women reported significant lower levels of Hg than the inactive group.

Fe toenail contents found in this study are similar than findings in toenails of other western populations [59], with no differences among sexes, which is in agreement with previous findings [28]. Fe, in the form of haemoglobin, is essential in the transport of oxygen from the lungs to the tissues where it is required. A fall in the circulating haemoglobin concentration is associated with a reduction in oxygen carrying capacity and a decreased exercise performance [50].

The Cr toenail content in this study is within the range than the values obtained in nails of healthy individuals in Ireland [54]. Cr is an essential trace element necessary for normal carbohydrate, lipid, and protein metabolism and its deficiency is associated with type II diabetes mellitus [60]. The Co toenail contents in this study are similar to those found in nails of Irish healthy adults [54]. Co is an essential trace element in the composition of cobalamin or vitamin B_12_, vitamins that do play an important role in maintaining normal immune function and metabolic homeostasis in elderly people as well as in preventing many age-associated diseases [55,60,61].

### Strengths and limitations of the study

This is the first report regarding trace element concentrations in toenails of Spanish older adults. Moreover, this is the first approach to evaluate the effects of physical activity on trace element contents in nails. The main finding of the present study was that the trace element contents in women are markedly influenced by the degree of physical activity, whereas no significant influence was observed in men. Nevertheless, the present sample is limited to Spanish older adults and caution should be made in drawing conclusions about other ethnic and age groups. There are also difficulties in comparing results with those obtained by previous studies. Most of studies are performed in younger participants than the participants of the present study. A second problem is that the studies performed in Mediterranean areas are also very scarce and most of studies aimed to evaluate trace elements in exposed people or to study an element, mainly Se [62–64], Zn [27], and Hg [43] in relation with several diseases. A third limitation was the small size of the sample in which the trace elements were analysed, in spite that this sample size was enough for statistical analysis.

## Conclusions

The present data added new information on the trace element contents in toenails of healthy Spanish older adults. The concentration of trace elements was similar in both sexes except for Ca which were lower in women. The trace element contents in women’s toenails, but not in men, were markedly influenced by physical activity, with higher levels of Ca and Fe and lower Hg among active females.
